# Effect of Fiducial Marker Design (Shape, Size, and Configuration) on the Trueness and Precision of CBCT Marker-Geometry Reproduction for CBCT–Intraoral Scan Registration: An In Vitro Study

**DOI:** 10.3390/diagnostics16162652

**Published:** 2026-08-20

**Authors:** Donghyo Yang, Gahyun Kim, Seoyun Hwang, Yo-Seob Seo, Joo-Hun Song

**Affiliations:** College of Dentistry, Chosun University, Gwangju 61452, Republic of Korea

**Keywords:** cone-beam computed tomography, fiducial markers, image registration, intraoral scanning, edentulous jaw, dental implants, trueness, precision

## Abstract

**Background/Objectives:** Cone-beam computed tomography (CBCT)-to-intraoral-scan registration in edentulous implant planning relies on radiopaque fiducial markers, yet how marker design governs marker-geometry reproduction is unclear. We quantified the effects of shape, size and configuration and where the error arose. **Methods:** Eighteen edentulous mandibular denture designs (three shapes × three sizes, 2–4 mm × two configurations; five markers each) were printed and CBCT-imaged three times at 0.2 mm voxel. Distances, angles, a leave-one-marker-out (LOMO) error and a markerless control were analyzed at the design level (n = 18). **Results:** Acquisition reproducibility was voxel-limited (median 49 μm). Marker size governed trueness and precision (partial ω^2^_p_ 0.25–0.65), with no shape effect detected. The LOMO error fell with size (541 to 359 μm; *p* = 0.004), whereas the markerless control did not (*p* = 0.653); the size effect differed between approaches. Twenty percent of 2 mm markers exceeded the 300 μm threshold—unstable localization, not failed detection. In the markerless control, 6 of 270 markers (2.2%) exceeded 1 mm despite acceptable global surface fits. **Conclusions:** A satisfactory global surface-fit residual may conceal substantial local misregistration, whereas fiducial landmarks permit independent verification of alignment. Marker size most directly governed reproduction fidelity; ≥3 mm diameters are recommended for the rotationally symmetric geometries tested.

## 1. Introduction

In implant treatment planning for edentulous patients, cone-beam computed tomography (CBCT), which carries bone information, is aligned into a common coordinate system with an intraoral scan (IOS), which carries soft-tissue and prosthetic information, to design the surgical guide [1]. Because registration error propagates directly into implant position and angulation [2], how accurately the two datasets are superimposed governs the precision of the entire digital workflow.

In dentate arches, the residual tooth surfaces provide stable anatomical landmarks that allow best-fit surface registration of the two surfaces (CBCT and IOS) [1]. In edentulous arches, however, such fixed references are absent and the soft tissue is compressible and deformable, so the reliability of surface-based registration is reduced and alternative references are required [3]; soft-tissue-based registration has been proposed specifically because no teeth are available in such cases [4]. To overcome this, radiopaque fiducial markers are attached to a scan template or denture and used as common reference points for point-based registration of the two datasets [1,3]; recent in vitro work in edentulous arches has compared marker materials and imaging methods for exactly this purpose [5], and registration strategy remains an active question in current computer-assisted implant surgery [6]. Previous studies have reported that the number and spatial configuration of such fiducial markers affect registration error [7].

At the same time, the linear-measurement accuracy of CBCT itself is limited. Several studies have reported systematic under- or over-estimation of CBCT-derived distances [8], and a systematic review of linear measurements for presurgical implant planning found a comparably wide error range across units and protocols [9]; more recent work confirms that the magnitude depends on the acquisition mode as well as the unit [10], and measurement accuracy has been examined in relation to voxel size and object size, although a significant voxel-size effect is not always observed within the clinical range [11]. Small radiopaque structures in particular are susceptible to the partial-volume effect and to detection limits [12], so marker size and shape may plausibly govern how faithfully the marker is reproduced by CBCT. Nevertheless, studies that systematically compare how faithfully CBCT reproduces marker geometry according to marker shape, size, and configuration are scarce; most prior work reports only the final registration error and does not distinguish whether the error originates from CBCT image acquisition or from marker localization.

The accuracy of marker-based registration is ultimately bounded by how accurately and precisely CBCT reproduces the three-dimensional marker geometry (inter-marker distances and angles). Marker-geometry reproduction fidelity is therefore a necessary condition for registration accuracy, and if a particular marker design is reproduced and localized more reliably, this constitutes direct grounds for improving registration. The aims of this in vitro study were therefore: (1) to quantify the trueness and precision with which CBCT reproduces the inter-marker geometry (distances and angles) of fiducial markers across three shapes × three sizes × two configurations (18 designs); (2) to identify the optimal marker design; and (3) to distinguish whether the error originates from CBCT image acquisition or from marker localization. The null hypothesis was that marker shape, size, and configuration have no effect on the trueness and precision of CBCT marker geometry.

## 2. Materials and Methods

### 2.1. Study Design and Specimens

**Figure 1:**
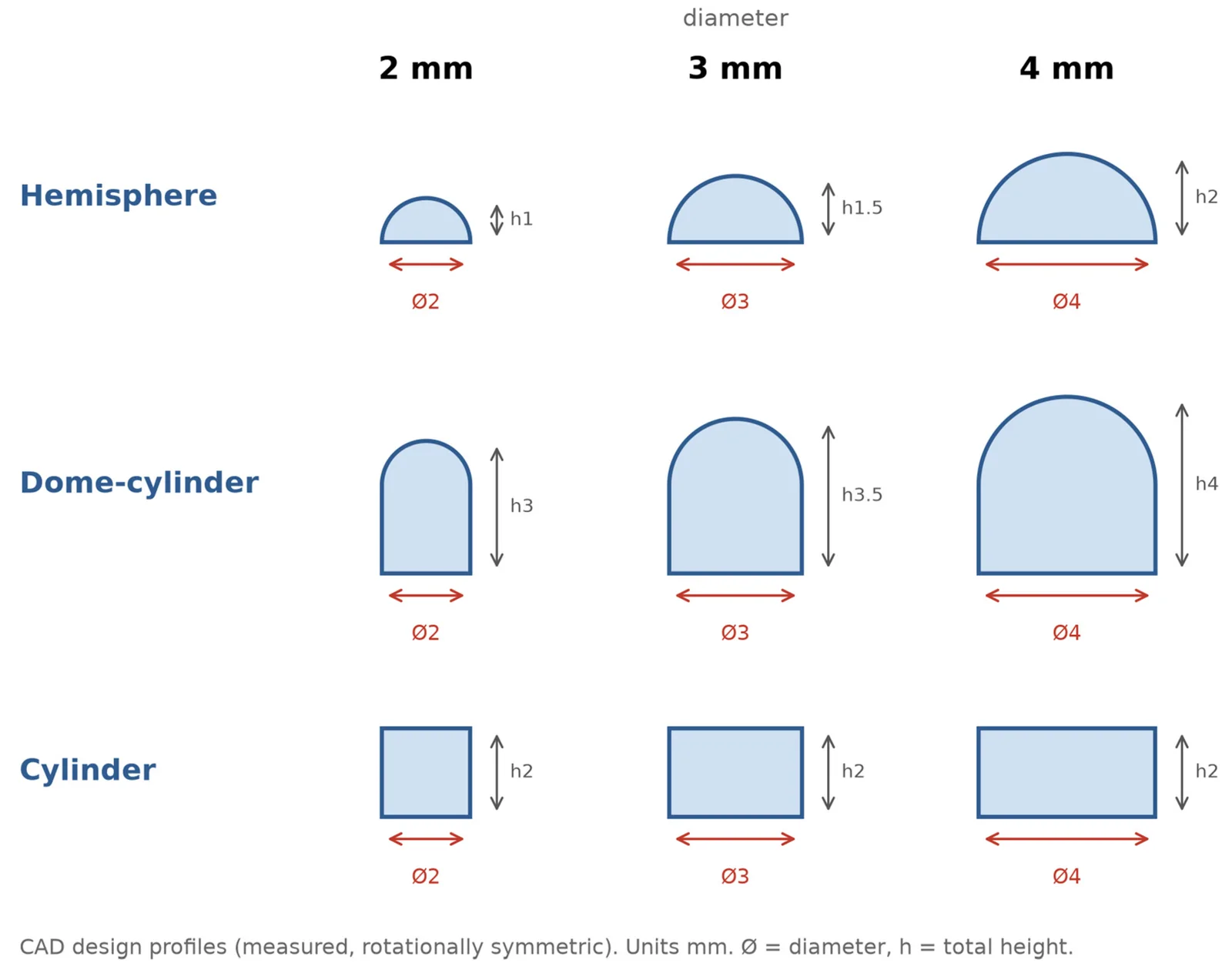
Fiducial marker shape and dimensions (CAD design profiles, rotationally symmetric). Hemisphere, dome-cylinder (cylinder + hemispherical dome), and cylinder at 2/3/4 mm diameter. Ø = diameter, h = total height.

**Figure 2:**
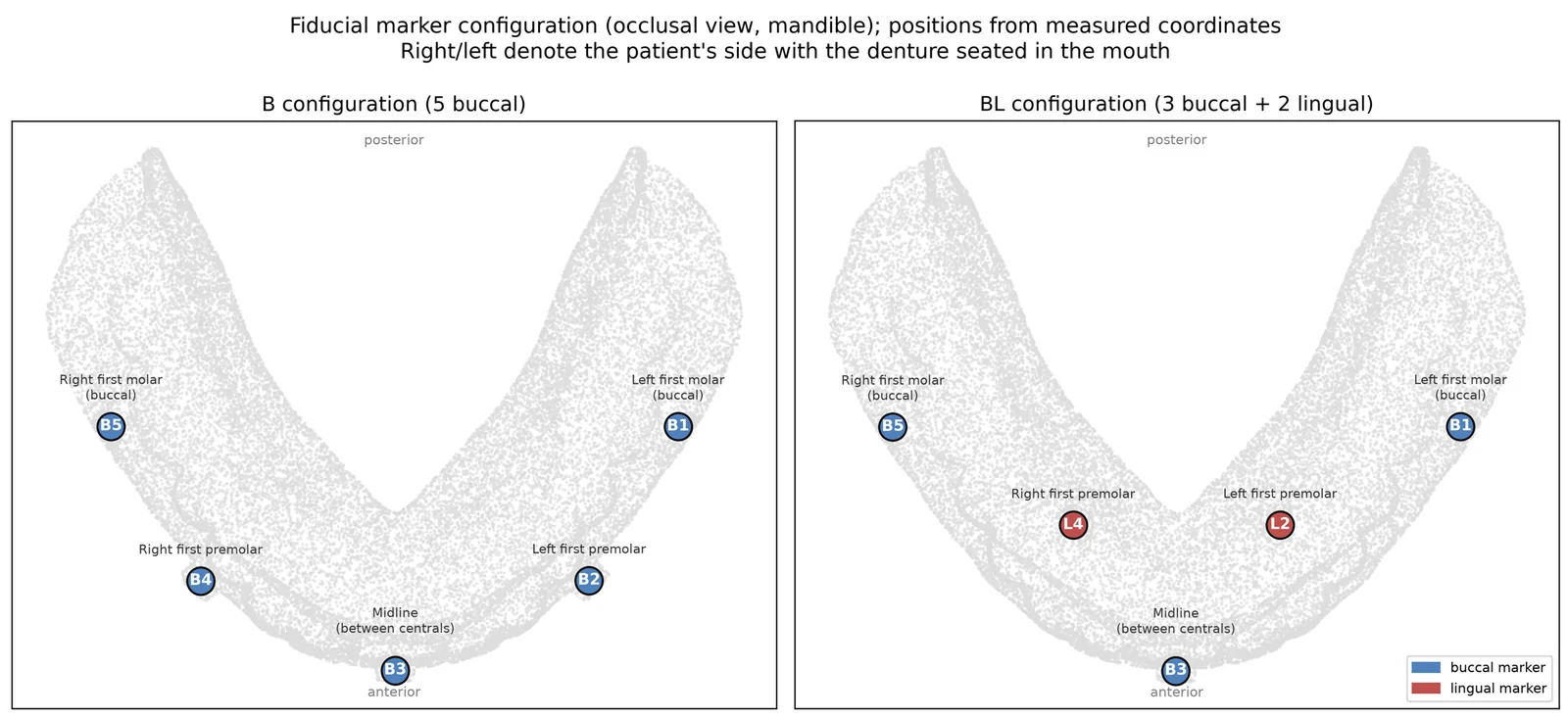
Fiducial marker configuration (occlusal view, mandible). Positions of the five markers for the B/BL configurations (measured coordinates). Buccal = blue, lingual = red. **Right** and **left** denote the patient’s side with the denture seated in the mouth, not the position of the panels.

The fiducial marker design comprised three factors: shape at three levels (hemisphere, dome-cylinder, cylinder), size at three levels (2, 3, and 4 mm diameter), and configuration at two levels (B: five buccal markers; BL: three buccal + two lingual markers). The lateral profile and measured dimensions of each shape are shown in Figure 1. The full factorial combination yielded 18 edentulous mandibular denture designs, each carrying the same five markers at anatomically defined fixed positions (Figure 2). The three shapes were chosen to represent hemispherical, domed, and cylindrical markers used clinically; the three sizes (2–4 mm) to span from near the detection limit to a clinically manageable range; and the two configurations to contrast buccal-only versus combined buccal–lingual clinical strategies. Marker positions were distributed widely along the dental arch following reproducible anatomical landmarks on the mandibular denture (first molar, first premolar, midline) to enhance registration. Marker positions also anticipated clinical translation, in which markers may be bonded directly to the mucosa of an edentulous ridge or carried on a denture base. The landmark available in a completely edentulous arch is the crest of the residual alveolar ridge, and the tissue that best supports bonding is the firm, keratinized masticatory mucosa overlying the ridge rather than the mobile lining mucosa of the vestibule. All markers were therefore placed on the buccal or lingual slope of the residual ridge, 1 mm below the ridge crest, keeping them on keratinized mucosa and away from movable vestibular tissue. Markers common to both configurations were (i) two at the mid-buccal aspect of each mandibular first molar, 1 mm below the ridge crest, and (ii) one at the midline of the anterior ridge, 1 mm below the crest. In configuration B, two markers were added on the buccal slope of the ridge at each first premolar position (1 mm below the crest); in configuration BL, two were added at the lingual aspect of the same positions. Laterality is described throughout with the denture seated in the mouth, so that right and left denote the patient’s right and left. Markers were thus arranged along the dental arch as left first molar (B1) → left first premolar (B2 or L2) → midline (B3) → right first premolar (B4 or L4) → right first molar (B5); B and BL differ only in whether the first-premolar markers are buccal or lingual.

**Figure 3:**
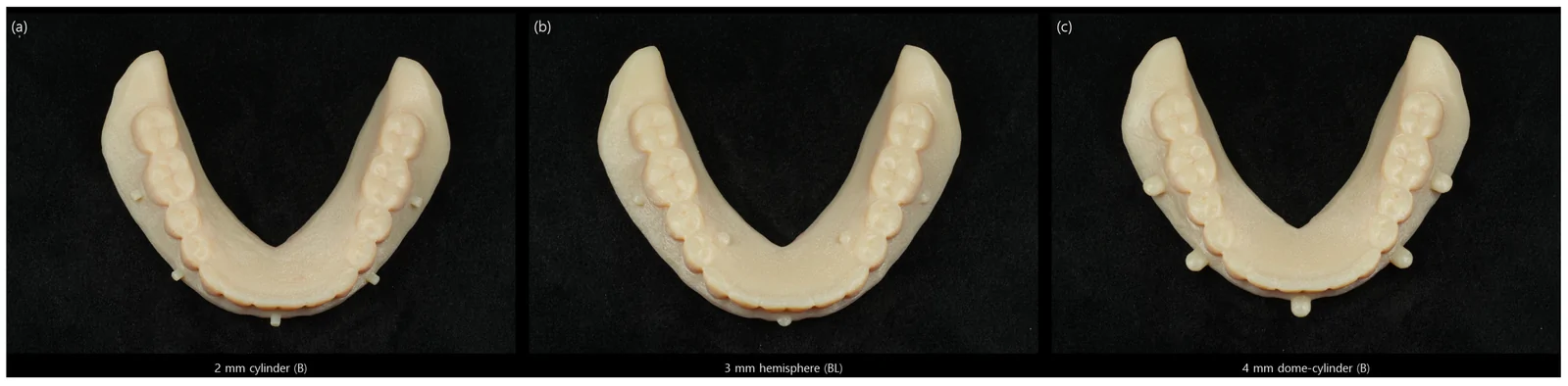
Representative printed specimens (occlusal view). (**a**) 2 mm cylinder, B; (**b**) 3 mm hemisphere, BL; (**c**) 4 mm dome-cylinder, B. See Figure 2 for marker positions.

**Figure 4:**
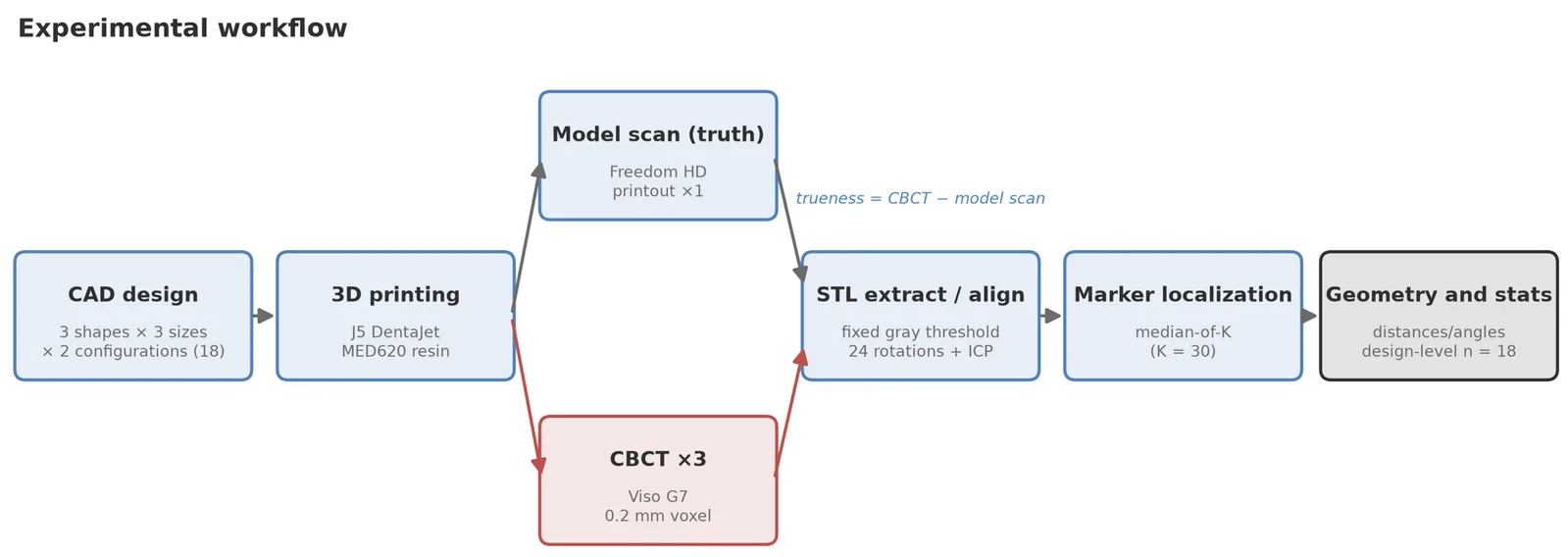
Overview of the experimental workflow: CAD design → 3D printing → model scan (ground truth) and CBCT ×3 → STL extraction/alignment → marker localization (median-of-K) → geometric measurement and design-level statistics. Red, the CBCT pathway; blue, the other workflow steps; gray, the final analysis output.

Specimens were printed by material-jetting (PolyJet) three-dimensional printing (J5 DentaJet, Stratasys, Minnetonka, MN, USA) using an opaque, rigid, biocompatible, A2-shade dental resin (MED620, Stratasys, Minnetonka, MN, USA) [13]. Representative printed specimens are shown in Figure 3, and representative specimens for each shape × size combination (configuration B) are compiled in Supplementary Figure S1. To incorporate and absorb the fabrication error of three-dimensional printing itself, ground truth was defined not as the design CAD but as the model scan of the actual printed specimen. Each specimen was scanned once with a laboratory model scanner (Freedom HD, DOF Inc., Seoul, Republic of Korea), which is specified by the manufacturer to an accuracy of approximately 10 μm and is assessed by the test methods for CAD/CAM digitizing devices given in ISO 12836 [14]. Because material-jetting printed models are reported to have dimensional accuracy on the order of tens of μm (e.g., PolyJet trueness ≈ 78 μm) [15]—nearly an order of magnitude coarser than the scanner—a single scan per specimen was adopted as ground truth. The overall experimental workflow is summarized in Figure 4.

### 2.2. CBCT Imaging

Each specimen was imaged three times with a CBCT unit (Planmeca Viso G7, Planmeca Oy, Helsinki, Finland). Acquisition settings were standardized: 80 kVp, 10 mA, 24.8 s exposure, 0.2 mm isotropic voxel size, and free field-of-view (FOV) mode. The repeated acquisitions were used to assess the reproducibility of CBCT image acquisition.

### 2.3. STL Extraction and Common-Frame Alignment

Each CBCT volume was segmented by gray-value thresholding. Because CBCT gray values are not calibrated to quantitative Hounsfield units, the surface-extraction threshold was determined once so that the extracted surface most closely matched a reference segmentation (a reference surface obtained with InVesalius 3.1; CTI Renato Archer, Campinas, Brazil) for this unit and protocol, and was then applied as a single fixed value to all scans. This fixed, deterministic thresholding excludes per-specimen manual adjustment and thereby removes observer variability at the segmentation step; because the identical threshold was applied to every scan, any threshold offset acts equally on all designs and cancels in the between-design comparisons. Faintly reconstructed marker regions were recovered by hysteresis-constrained region growing, and a triangular surface mesh (STL) was generated by marching cubes after Gaussian smoothing. The meshes generated by the model scanner were used directly as the reference meshes. Following mesh generation, alignment, localization, and measurement were performed using an identical pipeline for both imaging modalities to ensure that differences in processing methods did not confound the accuracy comparison. All extracted STLs were aligned to a common CAD coordinate frame using a robust alignment procedure. The procedure initialized the alignment with the 24 proper rotations of a cube to uniformly sample the orientation space and reduce convergence to local minima, followed by iterative closest point (ICP) refinement [16]. Because the 18 design CADs share identical coordinates, this CAD frame serves as the common reference frame for all specimens.

### 2.4. Marker Localization

Canonical seed positions (B1–B5, L2, L4) were defined once from the CAD. At each seed, the CAD model of the corresponding marker was fitted to the scan surface by ICP, and the known ground center (the geometrically defined marker center) of the fitted CAD (sphere center for the hemisphere and dome-cylinder; volume center for the cylinder) was computed. All three geometries are rotationally symmetric, so every principled center lies on the axis of symmetry, and for the cylinder the volume centroid, the bounding-box center and the midpoint of the maximum cross-sectional area coincide, the vertex centroid differing by only 7–14 μm (Table S2). The same definition was applied to the CBCT surface and to the model scan through one pipeline, so a systematic offset from the choice of center cancels in the trueness difference and leaves the repeat-to-repeat standard deviation, and hence precision, unchanged. To mitigate local-minimum convergence failures that can occur for small markers, the seed was perturbed randomly by ±0.4 mm and K = 30 independent restarts were run; the coordinate-wise median of the candidate centers (median-of-K consensus) was used as the final center [17]. The number of restarts was determined by observing beforehand that the frame-invariant reproducibility of 2 mm markers no longer improved and stabilized at K = 30 (identical at K = 40 and 50), consistent with the view that multi-start optimization has no universal constant and should be tuned to problem difficulty [17]. Minimum-residual selection (best-of-K) was rejected because it tended to select outliers for small markers. The fit residual (fit-RMS) was recorded as a quality-control index. This localization procedure was applied identically to CBCT and model scans so that any method-dependent systematic bias would cancel in the accuracy comparison (CBCT − model scan). In a sensitivity analysis, the median-of-K consensus only mitigated unstable localization of small markers relative to single fitting and did not change the ranking or significance conclusions of the design factors.

### 2.5. Geometric Measurements and Outcomes

From the five markers of each specimen, 10 inter-marker distances (_5_C_2_) and 30 triangle interior angles (_5_C_3_ × 3) were computed. Distances and angles are frame-invariant to rigid translation and rotation and are therefore unaffected by coordinate-alignment error. Trueness was defined as the difference between the mean of the three CBCT acquisitions and the model scan (ground truth), reported as signed bias, mean absolute error (MAE), and root-mean-square error (RMS) [18]. Precision was defined as the standard deviation across the three CBCT repetitions. Angles were represented identically in degrees (°).

### 2.6. CBCT Acquisition Reproducibility (Localization-Independent)

To assess pure image-acquisition reproducibility independent of marker localization, the three CBCT surfaces of each specimen were rigidly registered to one another, and inter-scan surface deviation was quantified as the exact point-to-mesh distance. Nearest point-to-point distance was not used because it is overestimated by the sampling spacing of the points.

### 2.7. Scale Handling

Because CBCT-derived distances showed a proportional deviation from the reference, an intercept-inclusive least-squares regression (L_CT = a + b·L_MS) was used to estimate the global scale b, and a single global b was applied identically to all designs for correction (no per-group scale was used, so that between-group comparisons were not contaminated by the correction itself). Results were reported in three tiers (as-measured/scale decomposition/scale-corrected residual) following ISO 5725 [18].

### 2.8. Statistical Analysis

The experimental unit was set to the physical denture model (n = 18) to structurally exclude the pseudo-replication that would arise from treating the many distances and repeated acquisitions of a single model as independent samples. After reducing each design to one summary value, factorial analysis of variance (log-transformed) with main effects and a shape × size interaction, together with the non-parametric Kruskal–Wallis test, was performed, and Tukey post hoc tests were applied to significant factors. Because there was one observation per cell (n = 1), the three-way interaction could not be tested; the model therefore included only the shape × size interaction, and configuration was evaluated as a main effect. The magnitude of each effect was quantified with partial eta-squared (η^2^_p_) and with partial omega-squared (ω^2^_p_), which corrects the upward bias of η^2^_p_ in small samples, and 90% confidence intervals (CIs) from the noncentral F distribution were reported (by convention, effect-size CIs use 90% rather than 95%). Effect sizes were interpreted using Cohen’s benchmarks (η^2^_p_ ≈ 0.01 small, 0.06 medium, ≥0.14 large). Residual normality of the factorial model was verified by the Shapiro–Wilk test. The significance level was α = 0.05. Statistics were performed in Python 3.13.14 (statsmodels 0.14.6, NumPy 2.4.6, SciPy 1.17.1). Generative AI-based tools were used as a coding assistant in implementing the analysis pipeline described in Section 2.3, Section 2.4, Section 2.5, Section 2.6, Section 2.7 and Section 2.8 (image segmentation, surface [STL] extraction, marker localization, and statistical analysis). All algorithms are deterministic and are specified in full in the preceding sections; no machine-learning model was used for segmentation, registration, or measurement. All AI-assisted code and outputs were reviewed, verified, and validated by the authors.

Two further metrics—the leave-one-marker-out (LOMO) validation error and the markerless surface-registration residual—were added after peer review and are therefore reported as post hoc secondary analyses rather than pre-planned confirmatory ones. They were analyzed within the same design-level framework: each design was first reduced to a single summary value (the median over its three acquisitions and its five folds or markers), giving an n = 18 vector, and marker-level distributions are reported only as secondary descriptive information. Because both metrics were obtained from the same 18 designs, the two values per design are paired; the method × size comparison was therefore fitted as a linear mixed-effects model with design as a random intercept and method and size as fixed effects, with a Kruskal–Wallis test on the within-design paired difference as an assumption-light cross-check. Being secondary and exploratory in nature, these analyses are interpreted through the direction and magnitude of the effects rather than through significance thresholds alone.

### 2.9. Leave-One-Marker-Out Validation

To relate marker-geometry reproduction to point-based registration, a leave-one-marker-out (LOMO) procedure was applied to every design and acquisition. Four of the five marker centers were used to compute a rigid point-based registration of the CBCT marker set onto the corresponding model-scan marker set by singular-value decomposition, and the Euclidean distance between the predicted and the measured position of the held-out fifth marker was recorded. The procedure was cycled over all five markers, and the resulting fifteen residuals per design (five folds × three acquisitions) were reduced to the design median. The held-out residual is a validation error evaluated at a marker position; it is not the target registration error at a clinical implant site.

### 2.10. Markerless Surface-Registration Control

A markerless control was performed on the same specimens and datasets. No marker information entered any stage of this control. A single common mask was defined once from the union of all seven candidate marker sites of the study design—the three positions shared by both configurations together with the buccal and lingual variants of the two remaining positions—and a fixed radius of 4.0 mm was applied at every site. The same mask was used for every design, every marker size and both modalities, so that the amount and spatial distribution of surface available for registration were identical throughout; this avoids the situation in which designs with different marker layouts would otherwise retain different amounts of usable surface.

Registration used the masked surfaces only. Approximately 45,000 points were sampled from the masked CBCT surface and 90,000 from the masked model-scan surface. The initial pose was derived from the principal axes of the two masked point clouds, with the four axis-sign ambiguities resolved by the iterative closest point score, so that neither the marker coordinates nor the marker-bearing common CAD frame contributed to initialization; the alignment was then refined by iterative closest point until the incremental translation fell below 1 μm or forty iterations were reached. The registration was verified to reproduce, to within approximately 26 μm at the marker positions, the result obtained when the same masked surfaces were registered from the common CAD frame. After registration, the marker centers—which took no part in computing the transform—served as independent verification targets, and the distance between the transformed CBCT marker center and the corresponding model-scan marker center was recorded. Residuals are additionally summarized as the number of markers exceeding a range of local-error thresholds.

## 3. Results

### 3.1. CBCT Acquisition Reproducibility

**Figure 5:**
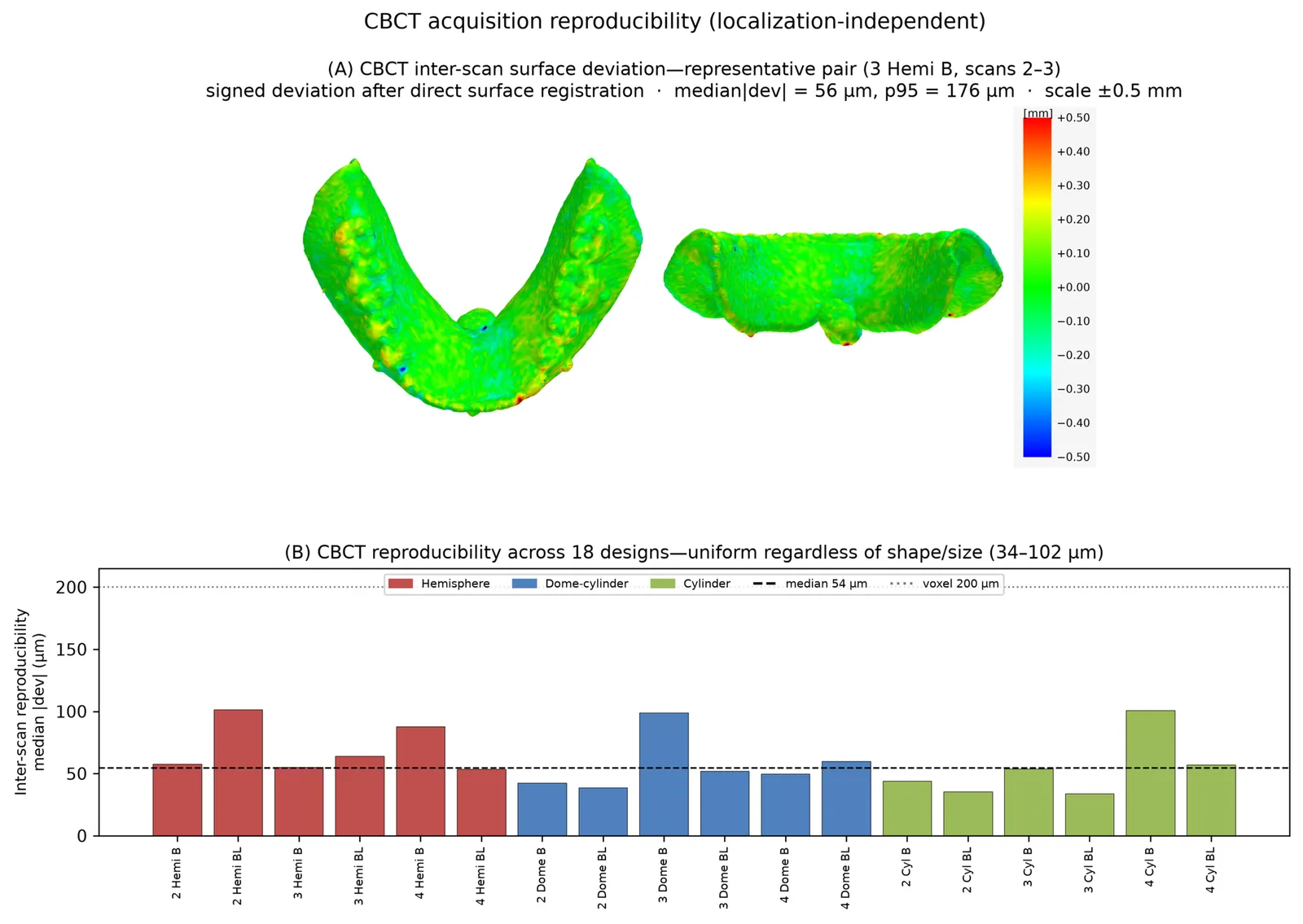
CBCT image-acquisition reproducibility (localization-independent). (**A**) Representative inter-scan surface deviation map; (**B**) uniformity across the 18 designs.

Inter-scan surface reproducibility, measured independently of marker localization, had a median of 49 μm (RMS 100 μm, 95th percentile 186 μm), about one-quarter of the voxel size (200 μm) (Figure 5, Table 1). This value was distributed within a narrow 31–112 μm range across all 18 designs, with no significant difference by marker shape (Kruskal–Wallis *p* = 0.069) or size (*p* = 0.239). The within-scan coefficient of variation of inter-marker distances was 0.28%, and the intraclass correlation coefficient was ICC(2,1) = 0.9997. CBCT image acquisition was highly reproducible down to the voxel limit, with consistent performance across all designs.

### 3.2. Marker Localization Quality

A marker center was obtained for all five markers in every specimen and acquisition (270/270, 100% detection); the stability of those centers is examined separately in Section 3.7. The median fit residual (fit-RMS) was 100 μm for model scans and 236 μm for CBCT, both at the voxel level (Table 2). The fit residual reflects the discrepancy between the ideal CAD shape and the actual printed and scanned marker surface (mainly three-dimensional printing deviation and surface roughness), rather than the uncertainty of the estimated marker center coordinate.

### 3.3. Inter-Marker Distance Trueness and Relative Scale

**Figure 6:**
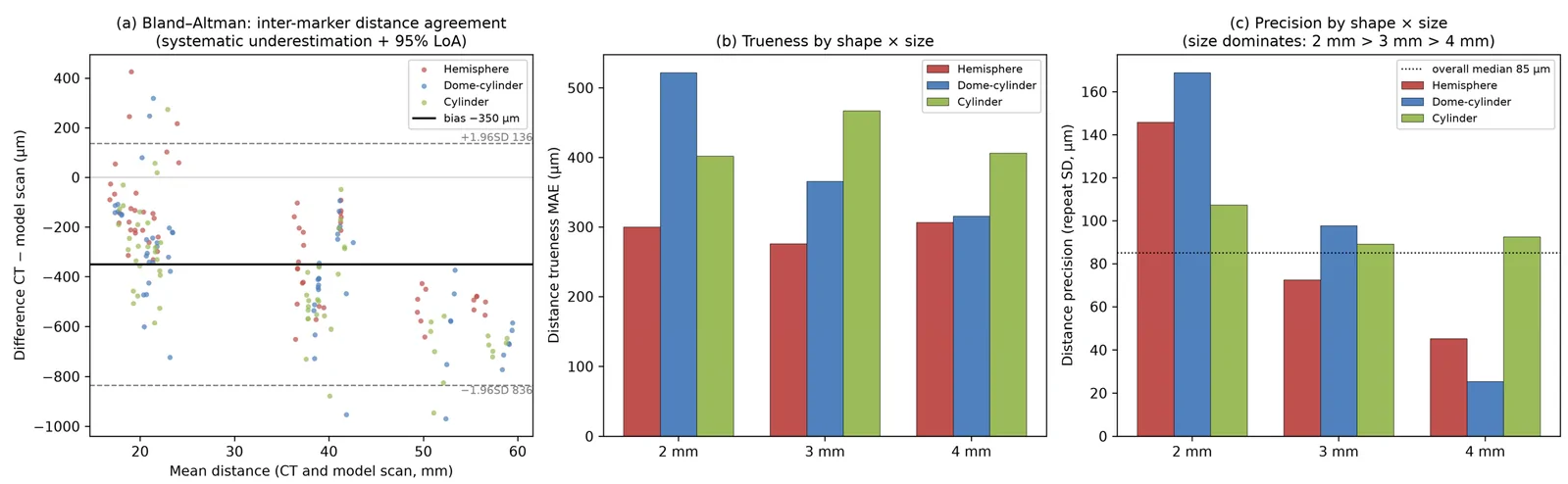
(**a**) Bland–Altman agreement of inter-marker distances (as-measured), showing a systematic underestimation with proportional bias; (**b**) as-measured distance trueness and (**c**) precision by shape × size. After global scale correction the apparent shape differences in trueness disappear (Table 3).

CBCT-derived distances tended to be proportionally contracted relative to the reference. In the intercept-inclusive regression, the slope was b = 0.9885 (95% CI 0.9864–0.9905, excluding 1) and the intercept was +41 μm (95% CI −35 to +118, not significant), indicating a uniform scaling effect without a constant offset (Figure 6, Bland–Altman [19]). Before correction, distance trueness was MAE 373 μm with a signed bias of −350 μm. Assigning separate scaling factors to each design suggested apparent differences among marker shapes, but these differences resulted from the positive intercept of hemispherical markers (+129 μm) being absorbed into the zero-intercept slope. In fact, the slopes of the three shapes were identical, and only the hemisphere showed a significant constant offset (+129 μm). The scale is therefore described as a common scale plus a hemisphere-specific offset.

**Table 3:** Inter-marker distance trueness (as-measured and scale-corrected).

Group	Bias (μm)	MAE Raw (μm)	RMS Raw (μm)	MAE Corrected (μm)
Overall	−350	373	429	146
Hemisphere	−257	294	342	155
Dome-cyl.	−380	401	454	146
Cylinder	−414	425	478	137
2 mm	−357	408	473	199
3 mm	−359	370	426	131
4 mm	−335	343	382	108
Buccal (B)	−421	422	481	118
Buccal + Lingual (BL)	−280	325	369	175

Global scale b = 0.9885 (95% CI 0.9864–0.9905), intercept +41 μm (n.s.). Corrected RMS 198 μm; leave-one-design-out out-of-sample MAE 148 μm. n.s. = not significant (*p* >= 0.05).

### 3.4. Distance Trueness After Scale Correction

When a single global scale factor (b) was applied identically to all designs (no per-group scale, so that between-group comparisons were not contaminated by the correction itself), distance trueness improved, with the MAE decreasing from 373 to 146 μm. In leave-one-design-out cross-validation—estimating the scale with one design excluded and applying it to the remaining designs—the out-of-sample MAE was 148 μm, essentially identical to the in-sample 146 μm, confirming no overfitting of the scale correction. After correction, MAE by shape was 137–155 μm (hemisphere 155, dome-cylinder 146, cylinder 137 μm) with no statistical difference, and by size 2 mm 199, 3 mm 131, 4 mm 108 μm, decreasing with increasing size (Table 3). Because inter-marker angles are frame-invariant and unaffected by scale, they showed essentially no bias relative to the reference (bias ≈ 0°), and angle trueness (MAE) was 0.56° overall. Angle trueness likewise improved with increasing size (2 mm 0.80°, 4 mm 0.41°), showing the same trend as distance, with no difference by shape (0.54–0.58°) and B (0.34°) superior to BL (0.78°) by configuration (Table 4).

### 3.5. Precision

Distance precision depended strongly on marker size: 2 mm 146 μm, 3 mm 85 μm, 4 mm 55 μm (overall median 84 μm). By configuration, BL (67 μm) was superior to B (114 μm). Angle precision was also size-dependent (2 mm 0.39° → 4 mm 0.18°, overall median 0.24°; Table 5). Precision, being a standard deviation, is unaffected by scale correction.

### 3.6. Statistical Comparison of Design Factors

In the design-level analysis of variance with the physical model as the experimental unit (n = 18), size showed a dominant effect on both scale-corrected distance trueness and precision (trueness *p* = 0.014, η^2^_p_ = 0.66, ω^2^_p_ = 0.43, 90% CI 0.13–0.77; precision *p* = 0.001, η^2^_p_ = 0.82, ω^2^_p_ = 0.65). Shape, in contrast, had a small effect size (trueness η^2^_p_ = 0.08, precision η^2^_p_ = 0.10, with bias-corrected ω^2^_p_ ≈ 0 in both cases) and was not statistically significant, consistent with a small real effect of shape. Configuration was significant for both measures (trueness *p* = 0.011, η^2^_p_ = 0.58, ω^2^_p_ = 0.36; precision *p* = 0.014, η^2^_p_ = 0.55, ω^2^_p_ = 0.33). In post hoc pairwise comparisons of size, 2 mm was significantly worse than 4 mm (*p* = 0.014), but no statistically significant difference was detected between 3 mm and 4 mm (*p* = 0.68). The direction of the configuration effect was opposite for the two measures: precision favored BL (67 vs. 114 μm), whereas scale-corrected trueness favored B (118 vs. 175 μm) (Table 6).

The shape × size interaction varied across outcome measures. For trueness it was not significant and appeared to be large (η^2^_p_ = 0.44), but after bias correction it decreased markedly to ω^2^_p_ = 0.11 with a 90% CI including zero (0.00–0.54); it should therefore be interpreted cautiously because of the limits of statistical power, with the possibility that η^2^_p_ is overestimated in a small sample. For precision, by contrast, the interaction was significant (*p* = 0.021, η^2^_p_ = 0.73, ω^2^_p_ = 0.49, 90% CI 0.12–0.78), and the CI excluded zero even after bias correction. Because each cell contains a single observation, this interaction is reported as a preliminary, hypothesis-generating trend that requires confirmation in replicated, adequately powered studies.

An exploratory examination of the shape-specific values within each size level (no inferential test; cell n = 1) showed that the relative ranking of shapes changed with size (Table 7, Figure 6). For scale-corrected distance trueness, the dome-cylinder showed greater error at 2 mm (226 μm), whereas the cylinder showed greater error at 3 mm (155 μm). At 4 mm, the difference between shapes was minimal (24 μm). For precision, the dome-cylinder exhibited greater variability at the smaller sizes, whereas the cylinder exhibited greater variability at 4 mm (84 vs. 24 μm for the cylinder and dome-cylinder, respectively). This size-dependent reversal of shape ranking corresponds to the significant shape × size interaction in precision. For angles the interaction was not significant.

Angles also showed a large size effect, mirroring distance (Table 6). Angle precision was significant for size (*p* = 0.026, η^2^_p_ = 0.60, ω^2^_p_ = 0.35), whereas angle trueness was marginally non-significant for size (*p* = 0.060) with a large effect size (η^2^_p_ = 0.50, ω^2^_p_ = 0.25). This size effect on angle trueness did not reach statistical significance because of limited power, but was suggestive given the monotonic trend (2 mm 0.80° → 4 mm 0.41°) and large effect size; it is interpreted cautiously because η^2^_p_ may be overestimated in a small sample. Shape was again small for angles (trueness η^2^_p_ = 0.10, precision η^2^_p_ = 0.34) and not significant. Configuration was significant for angle trueness (*p* = 0.004, η^2^_p_ = 0.67, ω^2^_p_ = 0.46), with B superior to BL (0.34° vs. 0.78°), consistent with the findings for distance trueness. The shape × size interaction seen for distance precision was not significant for angles (*p* = 0.780 and 0.875 for trueness and precision), and was thus confined to distance precision.

### 3.7. Localization Stability of 2 mm Markers

A marker center was obtained for every marker in every acquisition, so detection succeeded throughout (270/270); the difficulty with 2 mm markers was the stability of that center rather than a failure to detect it. The proportion of markers whose frame-invariant reproducibility exceeded 300 μm—approximately 1.5 voxels—was 20% (6/30) for 2 mm, versus 0% for 3 mm and 4 mm. This criterion was defined post hoc and was shown to be robust across thresholds from 200 to 400 μm: no 3 mm or 4 mm marker was classified as unstable at any threshold in that range (their maximum reproducibility values were 187 and 193 μm, respectively), whereas 2 mm markers exceeded every threshold tested (47%, 27%, 20%, 10% and 10% at 200, 250, 300, 350 and 400 μm; Table S1). Excluding the unstable 2 mm markers, the precision of 2 mm (116 μm) still remained worse than that of 3 mm (85 μm) and 4 mm (55 μm); 2 mm markers were therefore both less stable and, when stable, less precise.

### 3.8. Distinguishing Error Sources: Acquisition vs. Localization

**Figure 7:**
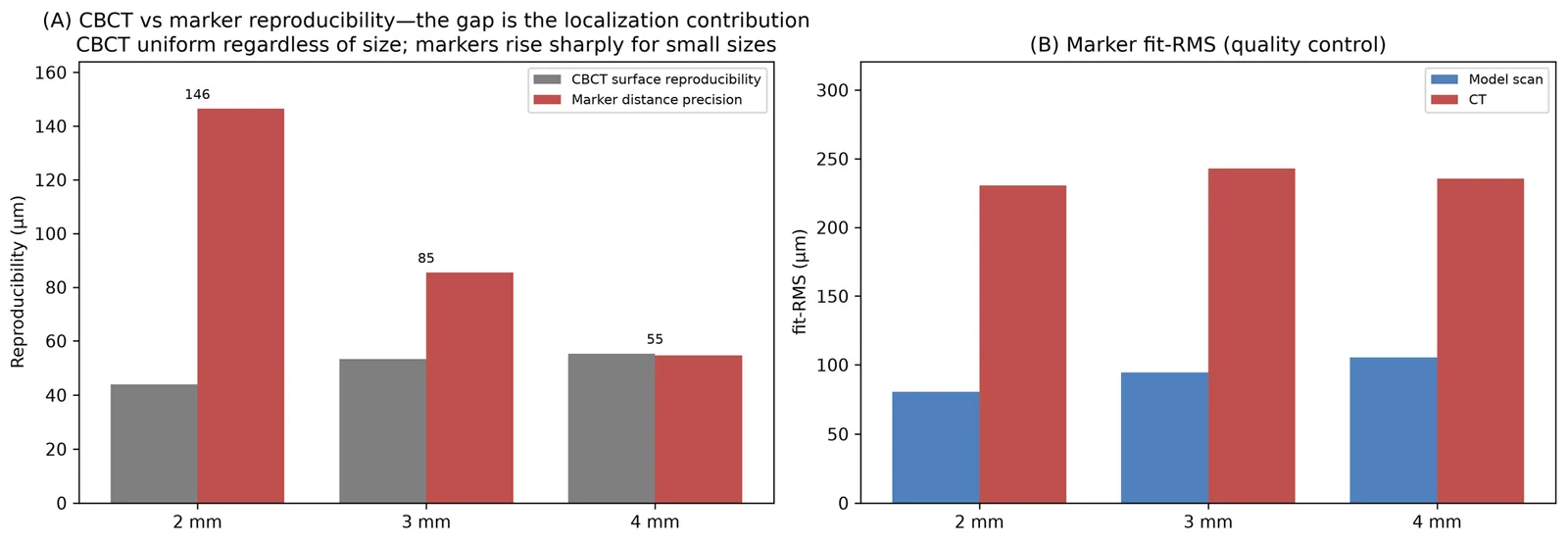
Contrast between CBCT surface reproducibility (uniform, size-independent) and marker measurement precision (sharply increasing for small markers): the bottleneck is localization.

Whereas CBCT surface reproducibility was uniform regardless of size (size-group median 44–55 μm: 2 mm 44, 3 mm 53, 4 mm 55), marker measurement precision improved markedly with increasing marker size (2 mm 146 μm, 4 mm 55 μm). The precision of 4 mm markers approached the level of CBCT acquisition reproducibility, whereas at 2 mm the gap was large (Figure 7).

### 3.9. Leave-One-Marker-Out Validation

The leave-one-marker-out validation error decreased with marker size (design-level medians 541, 454 and 359 μm at 2, 3 and 4 mm; *p* = 0.004, η^2^_p_ = 0.75, 90% CI 0.28–0.83, ω^2^_p_ = +0.56). No difference was detected between shapes (hemisphere 381, dome-cylinder 450, cylinder 519 μm; *p* = 0.309, η^2^_p_ = 0.25, 90% CI 0.00–0.48) or between configurations (*p* = 0.203); the numerically higher cylinder value therefore does not represent a detected effect. Design-level values are given in Table S3.

Two qualifications apply. First, the procedure is deliberately conservative: it uses four of the five markers and evaluates a point that took no part in the registration, so it is best read as a conservative validation surrogate rather than as a strict upper bound on the error of a registration that uses all five markers. Second, it is evaluated at a marker position and is not the clinical target registration error at an implant site, which depends on where the target lies relative to the fiducial array [20,21].

### 3.10. Markerless Surface Registration

Markerless registration on the masked surfaces was feasible, with a median point-to-mesh surface residual of 625 μm and a median residual at the marker positions of 363 μm at the design level. No size dependence was detected for the markerless residual (design-level medians 385, 382 and 350 μm at 2, 3 and 4 mm; *p* = 0.653), in contrast to the marker-based analysis above. Because both metrics come from the same 18 designs, this contrast was tested as a paired comparison rather than inferred from one test being significant and the other not. In the mixed-effects model with design as a random intercept, the method × size interaction was significant (χ^2^ = 10.78, df = 2, *p* = 0.005); the random-intercept variance, however, converged to essentially zero, and the assumption-light cross-check—a Kruskal–Wallis test on the within-design paired difference—did not reach the conventional threshold (*p* = 0.055). The most informative summary is therefore the paired ratio of the two residuals within each design, which fell monotonically with marker size (median marker-based/markerless ratio 1.34, 1.31 and 0.93 at 2, 3 and 4 mm). Taken together, the direction is consistent—the effect of marker size differed between the two registration approaches, being present for marker-based localization and absent for the markerless control—but the statistical evidence for that difference is moderate rather than strong.

The distribution of the markerless residuals, rather than their central tendency, is the clinically informative feature. Across all 270 markers, 40 residuals (14.8%) exceeded 500 μm, 13 (4.8%) exceeded 750 μm and 6 (2.2%) exceeded 1000 μm, with a maximum of 2847 μm (Table S5); the 1000 μm level is used here as a reference because reported CBCT–intraoral-scan registration errors and the deviations quoted for guided implant surgery are generally of sub-millimeter magnitude [22,23,24,25], so a local error beyond 1 mm would be clinically consequential. In the least favorable case, the global surface residual was 606 μm—an apparently acceptable surface fit—while the residual at a marker reached 2847 μm. A satisfactory global surface-fit residual can therefore conceal substantial local misregistration on these smooth, largely featureless edentulous surfaces, whereas fiducial markers, which take no part in the surface registration, permit that misalignment to be detected. A configuration effect was also present for the markerless residual (B 394 versus BL 327 μm; *p* = 0.013). This effect persisted after the common fixed mask was introduced, so it is not explained by differences in the surface available for registration; because the analysis uses no marker information, it cannot be a property of marker localization and is more plausibly attributable to the denture surfaces themselves. It is reported for completeness and is not interpreted as a marker-design finding. Design-level values and the exceedance counts are given in Tables S4 and S5.

## 4. Discussion

This study characterized, in a full factorial of shape, size, and configuration, how marker design affects the trueness and precision with which CBCT reproduces marker geometry for edentulous CBCT–IOS registration. The clearest finding is that CBCT image acquisition itself was not the predominant source of error. Acquisition reproducibility was only about one-quarter of the voxel size (49 μm) and uniform regardless of marker design, suggesting that the three repeated acquisitions adequately characterized acquisition precision under the present protocol. The design-to-design differences observed in marker-geometry measurement therefore originate from marker localization rather than from the reconstruction limit of CBCT. Because ground truth was the model scan of each printed specimen, three-dimensional printing error is absorbed into the reference rather than inflating the measured error, and the three repeated acquisitions isolate acquisition error from design effects.

This perspective explains the size effect. After scale was removed, both the trueness and precision of marker geometry were determined by size, whereas no statistically significant difference by shape was detected. The size effect showed a consistent monotonic trend across all three shapes and both configurations and is therefore unlikely to reflect specimen-to-specimen fabrication variability. The smaller the marker, the fewer voxels compose its surface and the less stable the CAD fit becomes; for 2 mm markers this manifested not merely as reduced precision but as unstable localization in 20% of markers. Indeed, acquisition reproducibility was uniform regardless of size, while marker measurement precision rose sharply for small markers, and the precision of 4 mm markers approached the level of acquisition reproducibility. In other words, sufficiently large markers allow localization to exploit most of the reproducibility that CBCT provides, whereas 2 mm markers do not. For precision a shape × size interaction reached significance in the present data; because each cell contains a single observation, we regard it as a preliminary, hypothesis-generating trend that requires confirmation in replicated, adequately powered studies rather than an established effect. On that basis the interaction was observed (bias-corrected ω^2^_p_ = 0.49, 90% CI excluding zero), suggesting that although the main effect of shape is small, the magnitude of the size benefit to precision may vary somewhat by shape. The non-significant main effect of shape is also explained by this interaction: because the size at which each shape shows relatively larger error differs (e.g., dome-cylinder at 2 mm, cylinder at 3 mm for distance trueness), these opposing shape rankings may be obscured when data are pooled across sizes. That is, “shape does not matter” is a pooled-level conclusion, and shape-specific variation is real for small markers. Exploratorily, the positive localization offset of the hemisphere was largest at small sizes (2 mm +175 → 4 mm +89 μm), consistent with the dome signal of small hemispheres being relatively more buried in the surrounding curvature. However, these are all exploratory observations with one observation per cell, so whether marker shape modifies localization performance at smaller marker sizes should be verified in future studies with replicated samples.

### 4.1. Marker Size and Detectability

The reduced localization performance of small radiopaque markers may be explained by the partial-volume effect and by detection limits. As the object size approaches the voxel size, signal is mixed in the boundary voxels and the surface is blurred [12], which destabilizes the basis of the CAD fit for small markers whose surface is composed of few voxels. A 2 mm-diameter marker spans only about 10 voxels in one direction at a 0.2 mm voxel, giving insufficient surface information; this is consistent with the observation that the size of the target structure governs the number of surface voxels and thereby affects measurement stability [12].

Clinically, taken together, these findings suggest that shape is not a decisive factor and provide grounds for recommending markers ≥ 3 mm in diameter. Because no statistically significant difference was detected between 3 mm and 4 mm, 4 mm is not strictly necessary, and 2 mm is unsuitable because a high proportion of its markers were unstably localized. In practice, placing radiopaque markers ≥ 3 mm in diameter on the scan template or denture yields stable CBCT reproduction of marker geometry and can contribute to more reliable subsequent point-based registration and to the surgical guides and navigation derived from it. The fact that the bottleneck of error lies in localization rather than acquisition implies that, under the present protocol, choosing an appropriate marker size (≥ 3 mm) is a more accessible route to improved marker-geometry reproduction than refining the acquisition step; whether finer voxels would confer an additional benefit was not tested here.

With respect to marker configuration, BL—using buccal and lingual markers together—was advantageous for precision, whereas B—using buccal markers only—was advantageous for scale-corrected trueness, so the direction differed by measure. This suggests that lingual markers, which differ from buccal markers in imaging and localization conditions (depth, surrounding structures), may carry a systematic bias while being more stable in repeatability. Configuration choice is therefore a matter to be judged according to the registration method rather than presented as a single conclusion. These configuration observations were obtained in vitro on dry printed models and may not translate directly to the clinical setting: the tongue, the palatal vault and the salivary pellicle can all affect the accessibility, imaging and localization of markers, and the lingual markers of the BL configuration are the most exposed to those influences. In the registration-based leave-one-marker-out analysis, no configuration effect was detected at the design level (*p* = 0.203), so configuration is not presented here as a determinant of registration performance.

### 4.2. CBCT Geometric Accuracy and Relative Scale

In this study, inter-marker distances showed an approximately uniform relative scale of about 1% relative to the reference. Systematic under- or over-estimation of CBCT linear measurements has been reported repeatedly [26,27], and its magnitude and direction depend on the unit, voxel, and reconstruction algorithm [28]. The relative scale in this study (about −1%) falls within this range. In particular, Wikner et al. reported that linear measurement of CBCT virtual models systematically underestimated distances relative to a caliper reference (*p* = 0.032), consistent with the contraction direction observed here [29]; Mukhia et al. reported a mandible-model deviation of about 0.43 mm at a 0.2 mm voxel [30], and Mehdizadeh et al. reported an implant-site measurement deviation under 1 mm [31], all within a sub-millimeter range. However, because the observed scale represents a relative difference between CBCT and the model scanner, it is difficult to attribute the absolute source of the scale to one side. The positive constant offset of hemispherical markers (+129 μm) is additive rather than multiplicative and therefore reflects marker-center localization bias rather than scale.

### 4.3. Implications for Registration

Because such a relative scale is not absorbed by rigid registration, it can propagate into registration error. In point-based registration, the target registration error (TRE) increases the farther the target deviates from the centroid of the fiducial array and the poorer the geometry of the array [20]. Because the marker array in this study lies nearly in one plane (limited vertical leverage), the conditions for a similarity registration that would absorb the scale are poor; it is therefore safer to recognize and correct the CBCT unit’s scale separately rather than to remove it at the registration step, and it is advisable to characterize the machine-specific scale in advance in clinical practice. However, because this study did not measure the actual TRE, the above is a theoretical implication derived from the marker-geometry reproduction results and not direct evidence about registration accuracy itself.

Inter-marker distances and angles were both governed by size, so the conclusion of size dominance is supported consistently across the two geometric descriptors of the marker constellation. These quantities characterize the stability and fidelity of that constellation; they are not direct measurements of the translational and rotational error of the final CBCT–IOS registration, and the angular mean absolute error reported here (overall 0.56°) should not be read as an implant angular deviation or as a lower bound on the achievable accuracy of registration. Angular fidelity is nonetheless of interest because rotational error in guided implant surgery is amplified with distance from the reference, and apical deviation is reported to exceed coronal deviation in large-scale analyses of guided-surgery accuracy [2,22]. A registration-based counterpart to these geometric measures is provided by the leave-one-marker-out analysis (Section 3.9), which is evaluated at a marker position and remains a conservative surrogate rather than the clinical target registration error. More broadly, whereas previous work has evaluated CBCT–IOS registration accuracy by three-dimensional deviation, including markerless and automatic approaches [4,23,32], the present design isolates the upstream stage that constrains that accuracy—namely, how faithfully CBCT reproduces marker geometry.

### 4.4. Comparison with Previous Studies

Studies that evaluated the accuracy of marker-based CBCT–IOS registration in edentulous arches by three-dimensional deviation have generally reported sub-millimeter registration error [33]. Indeed, Biun et al. reported that marker-based registration was superior (0.19–0.21 mm for a full arch) to markerless registration (0.58–0.70 mm) [24]; Motel et al. reported 0.008–0.01 mm error for best-fit registration on teeth [25]; Yang et al. reported 0.20 mm for maxillary registration [34]; and Wu et al. reported an RMSE of 0.21–0.26 mm for CBCT–model registration [35], all reporting errors within the sub-millimeter range. The scale-corrected inter-marker distance error of this study (about 0.15 mm) is the upstream stage of such registration and serves as a conservative reference against which the fidelity of marker geometry can be judged. That is, no matter how sophisticated the registration algorithm, if the marker itself is inaccurately reproduced, registration accuracy is bounded accordingly.

This study varied shape, size, and configuration while fixing the marker material to an opaque resin widely used clinically, and showed that the physical size of the marker—one axis of detectability—governs reproduction accuracy. The finding that size is dominant over shape suggests that absolute size, together with material, should be a primary consideration in marker selection. Marker material and metal artifact have also been reported to affect localization and registration: Biun et al. found that activating a metal-artifact-reduction algorithm changed registration trueness by only 0.041 mm and advised against relying on it in the presence of restoration artifact [36]. By fixing the material to a single opaque resin, this study excluded such confounding and isolated the net effect of shape, size, and configuration.

The influence of the number and spatial distribution of fiducial points on registration error is well established in point-based registration theory, and the more widely fiducial points are distributed to well surround the target, the smaller the target registration error [21,37]. That the BL configuration using buccal and lingual markers together was advantageous for precision in this study may relate to lingual markers widening the spatial distribution of the array, but at the same time the localization bias of lingual markers was disadvantageous for trueness, so the net effect differed by measure.

The sensitivity of small-structure measurement accuracy to voxel size has been confirmed in several studies, and it improves as the voxel becomes smaller [12,38]. In particular, the direction of the error depends on structure size relative to the voxel: alveolar bone thicker than the voxel was overestimated, whereas structures approaching or below the voxel size were underestimated, and reducing the voxel from 0.40 to 0.25 mm substantially improved accuracy [38]. The 2 mm marker failures in this study suggest that the sampled voxels of small markers are insufficient even at a 0.2 mm voxel, and this problem is expected to worsen at coarser voxels. Ultimately, because such marker-reproduction error may propagate to the accuracy of the implant surgical guide [2], reliable marker design is a prerequisite for securing the precision of the digital edentulous implant workflow.

### 4.5. Limitations

This study has the following limitations. First, only one physical model was fabricated per design (n = 1 per cell), so the three-way interaction could not be tested, and the three repetitions are measurement replicates, not fabrication replicates. Moreover, because the design-level sample is limited to 18, the finding that no significant difference by shape was detected should be interpreted within the limits of statistical power and not as proof of equivalence among shapes (the effect size of shape was also small: partial η^2^_p_ = 0.08–0.10). In addition, because there is one observation per cell, the simple main effect of shape at each size level (e.g., shape comparison within 3 mm) cannot be tested statistically, so the size-stratified shape comparison (Table 7) is an exploratory description only, and the size dependence of the shape effect requires future studies with replicated samples. Second, a single CBCT unit, a single voxel size (0.2 mm), and a single model scanner were used, so the observed relative scale is specific to this combination, and its direction and magnitude may vary by unit and protocol. The gray-value threshold used for segmentation is likewise calibrated to this unit and protocol and would require recalibration on other systems. Also, the model scan used as ground truth was acquired only once per specimen, so the repeatability of the scanner itself was not assessed, and although the scanner is assessed by the ISO 12836 test methods for CAD/CAM digitizing devices [14], its absolute accuracy was not separately verified with a certified phantom in this study; however, because material-jetting printed models are reported to have dimensional accuracy on the order of tens of μm (e.g., PolyJet trueness ≈ 78 μm) [15], using the scanned printout as ground truth is reasonable. Third, regarding the nature of the scale, the following is stated explicitly: the CT-derived inter-marker distances showed a proportional deviation from the reference (b = 0.9885, 95% CI 0.9864–0.9905; intercept +41 μm, not significant), consistent with an approximately uniform relative scale, which was therefore corrected with a single global factor as a first-order approximation. Whether this scale originates from the CBCT unit or the model scanner, its spatial uniformity across the field of view, and its behavior along the axial direction could not be fully characterized from the near-planar marker array alone and warrant a certified dimensional phantom. Fourth, this study was conducted under in vitro conditions and does not include the influence of soft tissue, patient movement, or metal artifact; also, although localization was automated to minimize observer variability, the pipeline was operated by a single operator and interobserver reproducibility was not assessed. Fifth, marker-geometry reproduction fidelity is an upstream determinant of registration accuracy rather than a direct measure of the target registration error (TRE), and the degree to which marker-geometry error propagates into actual registration error was not measured in this study. Imaging of a certified dimensional phantom (to verify the source of the scale and axial behavior), direct quantification of TRE, and comparison of voxel-size dependence require future study. Sixth, the radiographic density of the marker material was not measured against a certified standard; the material is identified by its commercial designation (MED620, Stratasys) and was imaged with a single protocol, so the size recommendation presupposes a material of comparable radiopacity. Seventh, the 300 micrometer criterion used to classify localization stability was defined post hoc; its influence was examined over the range 200–400 μm (Table S1), but robustness outside that range was not assessed. Finally, the markerless control and the leave-one-marker-out analysis were performed on the same in vitro datasets and share their limitations; neither replaces a direct clinical measurement of the target registration error. The markerless control also has limits of its own: it was computed with one masking radius and one registration algorithm, its residual at the marker positions is a verification measure rather than a clinical target registration error, and the paired comparison against the marker-based analysis rests on 18 designs, so the difference in the size effect between the two approaches should be regarded as moderate evidence rather than a settled result.

## 5. Conclusions

For fiducial markers used in edentulous CBCT–IOS registration, CBCT image-acquisition reproducibility was uniform down to the voxel limit (49 μm) regardless of marker design. The observed marker-geometry reproduction error therefore originates from marker localization rather than from image acquisition, and localization was the immediate bottleneck under the protocol tested. Localization is itself shaped by voxel size, partial-volume effects, reconstruction, segmentation and smoothing, so this does not imply that the limitation is independent of image resolution. A further observation concerns how alignment is verified: in the markerless control a satisfactory global surface-fit residual could coexist with a local error above 1 mm at a marker position (6 of 270 markers, maximum 2847 μm), so a global best-fit value alone may not reliably verify CBCT–intraoral-scan alignment on smooth edentulous surfaces, whereas fiducial landmarks—which take no part in a surface registration—permit that alignment to be verified independently. This localization fidelity was determined by marker size—for inter-marker distances and angles (bias-corrected partial ω^2^_p_ = 0.25–0.65) and likewise for the leave-one-marker-out validation error (*p* = 0.004)—with no statistically significant effect of shape detected among the three rotationally symmetric geometries examined. A shape × size interaction reached significance for distance precision but is regarded as a preliminary trend requiring replication, and it does not alter the size-based recommendation. Under the present acquisition and localization protocol, 2 mm markers showed limited suitability because 20% of them exceeded the 300 micrometer reproducibility threshold, indicating unstable localization rather than failed detection. Markers of at least 3 mm in diameter are therefore recommended for the geometries tested; no statistically significant difference was detected between the 3 mm and 4 mm groups, which suggests no clear additional benefit of increasing the diameter from 3 to 4 mm under these conditions. This recommendation is specific to the conditions studied—the CBCT unit used, a 0.2 mm voxel, the MED620 material, the fixed-threshold segmentation and the median-of-K localization—and presupposes a material of adequate radiopacity; it is not offered as a universal clinical threshold. It is also restricted to rotationally symmetric markers: non-symmetric or prismatic designs, which present flat faces, edges and corners and might support additional positional or orientational verification, were not evaluated and warrant separate study. Configuration effects differed in direction between measures—buccal–lingual was slightly better for precision and buccal-only for trueness—whereas no configuration effect was detected in the registration-based analysis, so configuration should be chosen according to the registration method rather than on the basis of these results alone. Finally, an approximately 1% relative scale was present in inter-marker distances and should be taken into account in marker-based registration; the extent to which marker-geometry error propagates into the actual target registration error requires future study.

## Figures and Tables

**Table 1:** CBCT acquisition reproducibility (inter-scan surface deviation, localization-independent).

Group	Median |Dev| (μm)	RMS (μm)	95th Pct (μm)
Overall	49	100	186
Hemisphere	57	110	203
Dome-cyl.	48	95	172
Cylinder	44	90	156
2 mm	44	77	146
3 mm	53	109	203
4 mm	55	104	199

CV 0.28%, ICC(2,1) = 0.9997. No shape/size dependence (*p* = 0.069/0.239). Voxel 200 μm.

**Table 2:** Marker localization quality (fit-RMS) and success rate.

Group	Model Scan (μm)	CBCT (μm)	Success
Overall	100	236	100%
Hemisphere	92	228	100%
Dome-cyl.	90	219	100%
Cylinder	112	266	100%
2 mm	80	230	100%
3 mm	94	243	100%
4 mm	106	236	100%

fit-RMS = CAD-fit surface residual (quality control), not center uncertainty. Success 270/270 (CBCT).

**Table 4:** Inter-marker angle trueness by group (scale-invariant; CBCT − reference, degrees).

Group	Bias (°)	MAE (°)
Overall	0.0	0.56
Hemisphere	0.0	0.58
Dome-cyl.	0.0	0.54
Cylinder	0.0	0.57
2 mm	0.0	0.8
3 mm	0.0	0.47
4 mm	0.0	0.41
Buccal (B)	0.0	0.34
Buccal + Lingual (BL)	0.0	0.78

Angles are frame- and scale-invariant (no scale correction). Size trend 2 mm 0.80° → 4 mm 0.41°; configuration B 0.34° vs. BL 0.78°; shape 0.54–0.58° (n.s.). n.s. = not significant (*p* >= 0.05).

**Table 5:** Inter-marker distance and angle precision (SD across three CBCT scans).

Group	Distance Precision (μm)	Angle Precision (°)
Overall	84	0.24
2 mm	146	0.39
3 mm	85	0.23
4 mm	55	0.18
Buccal (B)	114	0.3
Buccal + Lingual (BL)	67	0.19
Hemisphere	72	0.27
Dome-cyl.	92	0.2
Cylinder	84	0.31

Precision (SD) is scale-invariant. Size dominates (2 vs. 4 mm *p* = 0.006).

**Table 6:** Design-level effect sizes (n = 18): *p*; partial η^2^_p_ (90% CI); bias-corrected ω^2^_p_—distance and angle.

Effect	Distance Trueness	Distance Precision	Angle Trueness	Angle Precision
Shape	*p* 0.732 n.s.; η^2^_p_ 0.08 [0.00,0.27]; ω^2^_p_ −0.08	*p* 0.649 n.s.; η^2^_p_ 0.10 [0.00,0.32]; ω^2^_p_ −0.06	*p* 0.665 n.s.; η^2^_p_ 0.10 [0.00,0.31]; ω^2^_p_ −0.07	*p* 0.186 n.s.; η^2^_p_ 0.34 [0.00,0.55]; ω^2^_p_ +0.11
Size	*p* 0.014 *; η^2^_p_ 0.66 [0.13,0.77]; ω^2^_p_ +0.43	*p* 0.001 **; η^2^_p_ 0.82 [0.42,0.87]; ω^2^_p_ +0.65	*p* 0.060 n.s.; η^2^_p_ 0.50 [0.00,0.66]; ω^2^_p_ +0.25	*p* 0.026 *; η^2^_p_ 0.60 [0.06,0.73]; ω^2^_p_ +0.35
Configuration	*p* 0.011 *; η^2^_p_ 0.58 [0.11,0.74]; ω^2^_p_ +0.36	*p* 0.014 *; η^2^_p_ 0.55 [0.09,0.73]; ω^2^_p_ +0.33	*p* 0.004 **; η^2^_p_ 0.67 [0.21,0.80]; ω^2^_p_ +0.46	*p* 0.111 n.s.; η^2^_p_ 0.29 [0.00,0.56]; ω^2^_p_ +0.11
Shape × Size	*p* 0.272 n.s.; η^2^_p_ 0.44 [0.00,0.54]; ω^2^_p_ +0.11	*p* 0.021 *; η^2^_p_ 0.73 [0.12,0.78]; ω^2^_p_ +0.49	*p* 0.780 n.s.; η^2^_p_ 0.18 [0.00,0.25]; ω^2^_p_ −0.14	*p* 0.875 n.s.; η^2^_p_ 0.13 [0.00,0.16]; ω^2^_p_ −0.19

Physical model as unit, log-transformed. Distance trueness = scale-corrected MAE; angle needs no scale correction. η^2^_p_ is upward-biased in small samples → partial ω^2^_p_ shown (negative ⇒ ≈0). 90% CI from noncentral F. Cohen: η^2^_p_ ≈ 0.01/0.06/0.14 = small/medium/large. Size effect large across all four measures; shape small (ω^2^_p_ ≈ 0); configuration significant for distance and angle trueness; shape × size significant only for distance precision. n.s. = not significant (*p* >= 0.05); * *p* < 0.05; ** *p* < 0.01.

**Table 7:** Exploratory shape × size cell means (configuration-pooled). No inferential test—cell n = 1.

Measure	Size	Hemisphere	Dome-Cyl.	Cylinder	Range (Worst)
Trueness MAE (μm)	2 mm	212	226	160	66 (Dome-cyl.)
	3 mm	133	105	155	50 (Cylinder)
	4 mm	120	108	96	24 (Hemisphere)
Precision (μm)	2 mm	150	165	113	52 (Dome-cyl.)
	3 mm	73	94	79	21 (Dome-cyl.)
	4 mm	39	24	84	60 (Cylinder)

Exploratory/descriptive only (cell n = 1, no inferential statistics). Trueness = scale-corrected MAE, precision = median SD. The worst-performing shape changes with size (trueness: dome-cylinder at 2 mm, cylinder at 3 mm, near-equal at 4 mm; precision: dome-cylinder at small sizes, cylinder at 4 mm), so shape rankings do not persist across sizes—the descriptive basis for the shape × size interaction, which is regarded as preliminary in precision (Table 6) and for the non-significant shape main effect (rank reversals cancel when sizes are pooled). See also Figure 6.

## Data Availability

The minimal dataset supporting the conclusions of this article is included as a Supporting Information File. Further raw data are available from the corresponding author on request.

## Supplementary Materials

The following supporting information can be downloaded at https://www.mdpi.com/article/10.3390/diagnostics16162652/s1. Figure S1: Representative printed specimens for each shape × size combination (configuration B) photographed on a 1 mm grid; Table S1: Sensitivity of the localization-stability criterion to the reproducibility threshold; Table S2: Comparison of candidate center definitions for the cylinder (CAD geometry); Table S3: Design-level leave-one-marker-out (LOMO) validation error (n = 18); Table S4: Design-level markerless surface-registration residual at marker positions (n = 18); Table S5: Markerless residuals at marker positions exceeding local-error thresholds (270 markers).

## References

[1] Flügge T., Derksen W., te Poel J., Hassan B., Nelson K., Wismeijer D. (2017). Registration of cone beam computed tomography data and intraoral surface scans—A prerequisite for guided implant surgery with CAD/CAM drilling guides. Clin. Oral Implant Res..

[2] Khaohoen A., Powcharoen W., Sornsuwan T., Chaijareenont P., Rungsiyakull C., Rungsiyakull P. (2024). Accuracy of implant placement with computer-aided static, dynamic, and robot-assisted surgery: A systematic review and meta-analysis of clinical trials. BMC Oral Health.

[3] Lin C.-C., Wu C.-Z., Huang M.-S., Huang C.-F., Cheng H.-C., Wang D.P. (2020). Fully digital workflow for planning static guided implant surgery: A prospective accuracy study. J. Clin. Med..

[4] Deferm J.T., Nijsink J., Baan F., Verhamme L., Meijer G., Maal T. (2022). Soft tissue-based registration of intraoral scan with cone beam computed tomography scan. Int. J. Oral Maxillofac. Surg..

[5] Ressurreição Y.T.S.D., Bommarito R., Gomes R.D., Romano M.M., Costa C., Nishyama R., Mukai M.K. (2025). Influence of fiducial markers and imaging methods on registration accuracy in edentulous arches. J. Prosthet. Dent..

[6] Wang Y., Chen J., Zhao Y., Shi B., Zhang Y., Yan Q. (2026). Comparative accuracy of intraoral scanning and CBCT registration in robotic computer-assisted implant surgery for partially edentulous patients: A randomised controlled trial. J. Clin. Periodontol..

[7] Fan S., Hung K., Bornstein M.M., Huang W., Wang F., Wu Y. (2019). The effect of the configurations of fiducial markers on accuracy of surgical navigation in zygomatic implant placement: An in vitro study. Int. J. Oral Maxillofac. Implant.

[8] Lascala C.A., Panella J., Marques M.M. (2004). Analysis of the accuracy of linear measurements obtained by cone beam computed tomography (CBCT-NewTom). Dentomaxillofac. Radiol..

[9] Fokas G., Vaughn V.M., Scarfe W.C., Bornstein M.M. (2018). Accuracy of linear measurements on CBCT images related to presurgical implant treatment planning: A systematic review. Clin. Oral Implant Res..

[10] Kaaber L., Matzen L.H., Spin-Neto R., Schropp L. (2024). Low-dose, standard, and high-resolution cone beam computed tomography for alveolar bone measurements related to implant planning: An ex vivo study in human specimens. Clin. Oral Implant Res..

[11] Damstra J., Fourie Z., Huddleston Slater J.J.R., Ren Y. (2010). Accuracy of linear measurements from cone-beam computed tomography-derived surface models of different voxel sizes. Am. J. Orthod. Dentofac. Orthop..

[12] Maret D., Telmon N., Peters O.A., Lepage B., Treil J., Inglèse J.M., Peyre A., Kahn J.L., Sixou M. (2012). Effect of voxel size on the accuracy of 3D reconstructions with cone beam CT. Dentomaxillofac. Radiol..

[13] Rebong R.E., Stewart K.T., Utreja A., Ghoneima A.A. (2018). Accuracy of three-dimensional dental resin models: A comparative study of models created by fused deposition modeling, stereolithography, and Polyjet prototype technologies. Angle Orthod..

[14] (2015). Dentistry—Digitizing Devices for CAD/CAM Systems for Indirect Dental Restorations—Test Methods for Assessing Accuracy.

[15] Kim S.Y., Shin Y.S., Jung H.D., Hwang C.J., Baik H.S., Cha J.Y. (2018). Precision and trueness of dental models manufactured with different 3-dimensional printing techniques. Am. J. Orthod. Dentofac. Orthop..

[16] Besl P.J., McKay N.D. (1992). A method for registration of 3-D shapes. IEEE Trans. Pattern Anal. Mach. Intell..

[17] Martí R., Resende M.G.C., Ribeiro C.C. (2013). Multi-start methods for combinatorial optimization. Eur. J. Oper. Res..

[18] (2023). Accuracy (Trueness and Precision) of Measurement Methods and Results—Part 1: General Principles and Definitions.

[19] Bland J.M., Altman D.G. (1999). Measuring agreement in method comparison studies. Stat. Methods Med. Res..

[20] Fitzpatrick J.M., West J.B., Maurer C.R. (1998). Predicting error in rigid-body point-based registration. IEEE Trans. Med. Imaging.

[21] Hamming N.M., Daly M.J., Irish J.C., Siewerdsen J.H. Effect of fiducial configuration on target registration error in intraoperative cone-beam CT guidance of head and neck surgery. Proceedings of the 30th Annual International Conference of the IEEE Engineering in Medicine and Biology Society.

[22] Tahmaseb A., Wu V., Wismeijer D., Coucke W., Evans C. (2018). The accuracy of static computer-aided implant surgery: A systematic review and meta-analysis. Clin. Oral Implant Res..

[23] Ntovas P., Marchand L., Finkelman M., Revilla-León M., Att W. (2024). Accuracy of manual and artificial intelligence-based superimposition of cone-beam computed tomography with digital scan data, utilizing an implant planning software: A randomized clinical study. Clin. Oral Implant Res..

[24] Biun J., Dudhia R., Arora H. (2023). The in-vitro accuracy of fiducial marker-based versus markerless registration of an intraoral scan with a cone-beam computed tomography scan in the presence of restoration artifact. Clin. Oral Implant Res..

[25] Motel C., Kirschner C., Förtsch F., Buchbender M., Wichmann M., Matta R.E. (2025). The influence of the superimposition procedure and type of intraoral impression on the superimposition accuracy of CBCT scans with dental impressions in implant planning: An in-vitro study. Int. J. Implant Dent..

[26] Ballrick J.W., Palomo J.M., Ruch E., Amberman B.D., Hans M.G. (2008). Image distortion and spatial resolution of a commercially available cone-beam computed tomography machine. Am. J. Orthod. Dentofac. Orthop..

[27] Pauwels R., Araki K., Siewerdsen J.H., Thongvigitmanee S.S. (2015). Technical aspects of dental CBCT: State of the art. Dentomaxillofac. Radiol..

[28] Periago D.R., Scarfe W.C., Moshiri M., Scheetz J.P., Silveira A.M., Farman A.G. (2008). Linear accuracy and reliability of cone beam CT derived 3-dimensional images constructed using an orthodontic volumetric rendering program. Angle Orthod..

[29] Wikner J., Hanken H., Eulenburg C., Heiland M., Gröbe A., Assaf A.T., Riecke B., Friedrich R.E. (2016). Linear accuracy and reliability of volume data sets acquired by two CBCT-devices and an MSCT using virtual models: A comparative in-vitro study. Acta Odontol. Scand..

[30] Mukhia N., Birur N.P., Shubhasini A.R., Shubha G., Keerthi G. (2021). Dimensional measurement accuracy of 3-dimensional models from cone beam computed tomography using different voxel sizes. Oral Surg. Oral Med. Oral Pathol. Oral Radiol..

[31] Mehdizadeh M., Rostamzadeh P., Taheri H. (2023). Impact of using standard and high-resolution exposure modalities of cone-beam computed tomography (CBCT) system for dental implants dimension measurements. Adv. Biomed. Res..

[32] Zheng Q., Wu Y., Chen J., Wang X., Zhou M., Li H., Lin J., Zhang W., Chen X. (2025). Automatic multimodal registration of cone-beam computed tomography and intraoral scans: A systematic review and meta-analysis. Clin. Oral Investig..

[33] Farah R.I., Alresheedi B., Al-Haj Ali S.N. (2026). Validation of a novel implant-retained fiducial marker system for digital scan alignment in the edentulous mandible: An accuracy study. J. Prosthodont..

[34] Yang Q., Cai M., Hao S., Chu H., Wang Y., Hu M., Yang H., Wang Z. (2025). Accuracy and reliability of a three-dimensional superimposition method for maxillary jaws and dentition. BMC Oral Health.

[35] Wu D., Jiang J., Wang J., Zhou S., Qian K. (2024). Accuracy evaluation of dental CBCT and scanned model registration method based on pulp horn mapping surface: An in vitro proof-of-concept. BMC Oral Health.

[36] Biun J., Dudhia R., Arora H. (2024). The influence of metal artifact reduction on the trueness of registration of a cone-beam computed tomography scan with an intraoral scan in the presence of severe restoration artifact. J. Prosthodont..

[37] Zhang W., Wang C., Yu H., Liu Y., Shen G. (2011). Effect of fiducial configuration on target registration error in image-guided cranio-maxillofacial surgery. J. Craniomaxillofac. Surg..

[38] Sun Z., Smith T., Kortam S., Kim D.G., Tee B.C., Fields H. (2011). Effect of bone thickness on alveolar bone-height measurements from cone-beam computed tomography images. Am. J. Orthod. Dentofac. Orthop..

