# Supplementary material for: Effect of Fiducial Marker Design (Shape, Size, and Configuration) on the Trueness and Precision of CBCT Marker-Geometry Reproduction for CBCT–Intraoral Scan Registration: An In Vitro Study
Source: Diagnostics (Basel). 2026 Aug 20;16(16):2652. doi: 10.3390/diagnostics16162652 (PMC13512746; doi:10.3390/diagnostics16162652)
Supplement: Supplementary file 1 [file diagnostics-16-02652-s001.zip › Tables S1-S5.pdf]

Supplementary Materials

Effect of Fiducial Marker Design (Shape, Size, and Configuration) on the Trueness and Precision of CBCT Marker-Geometry Reproduction for CBCT–Intraoral Scan Registration: An In Vitro Study

Figure S1 is provided as a separate image file. Tables S1–S5 are given below.

Table S1. Sensitivity of the localization-stability criterion to the reproducibility threshold.

Marker size (mm)	Markers (n)	> 200 µm	> 250 µm	> 300 µm	> 350 µm	> 400 µm
2	30	14 (47%)	8 (27%)	6 (20%)	3 (10%)	3 (10%)
3	30	0 (0%)	0 (0%)	0 (0%)	0 (0%)	0 (0%)
4	30	0 (0%)	0 (0%)	0 (0%)	0 (0%)	0 (0%)

Frame-invariant reproducibility distribution by size (µm): 2 mm — median 191, 90th percentile 356, maximum 829; 3 mm — median 87, 90th percentile 159, maximum 187; 4 mm — median 64, 90th percentile 123, maximum 193.

The 300 µm criterion was defined post hoc. Across the range 200–400 µm no 3 mm or 4 mm marker was classified as unstable, because their maximum reproducibility values (187 and 193 µm) lie below the lowest threshold tested.

Table S2. Comparison of candidate center definitions for the cylinder (CAD geometry).

Diameter (mm)	Volume centroid vs vertex centroid (µm)	Volume centroid vs bounding-box center (µm)	Maximum axial difference (µm)
2	7.2	0	1.5
3	10.7	0	1.5
4	14.3	0	1.5

All principled center definitions for a rotationally symmetric cylinder lie on the symmetry axis. The volume centroid, the bounding-box center and the midpoint of the maximum cross-sectional area are identical; the vertex centroid differs by 7–14 µm, essentially radially. These differences are two orders of magnitude smaller than the reported measurement errors.

Table S3. Design-level leave-one-marker-out (LOMO) validation error (n = 18).

Shape	Size (mm)	Configuration	LOMO validation error, median (µm)
Hemisphere	2	B	650
Hemisphere	2	BL	1636
Hemisphere	3	B	323
Hemisphere	3	BL	390
Hemisphere	4	B	372
Hemisphere	4	BL	346

Dome-cylinder	2	B	541
Dome-cylinder	2	BL	515
Dome-cylinder	3	B	386
Dome-cylinder	3	BL	519
Dome-cylinder	4	B	271
Dome-cylinder	4	BL	325
Cylinder	2	B	541
Cylinder	2	BL	515
Cylinder	3	B	548
Cylinder	3	BL	523
Cylinder	4	B	400
Cylinder	4	BL	385

Each value is the median over the three acquisitions and the five leave-one-marker-out folds of that design. Group medians: 541, 454 and 359 μm at 2, 3 and 4 mm ($p = 0.004$); hemisphere 381, dome-cylinder 450, cylinder 519 μm ($p = 0.309$); B 400, BL 515 μm ($p = 0.203$).

Table S4. Design-level markerless surface-registration residual at marker positions (n = 18).

**Shape**	**Size (mm)**	**Configuration**	**Residual at markers, median (μm)**	**Maximum (μm)**	**Surface RMS (μm)**
Hemisphere	2	B	331	807	616
Hemisphere	2	BL	284	2847	599
Hemisphere	3	B	391	460	607
Hemisphere	3	BL	301	2437	623
Hemisphere	4	B	394	796	680
Hemisphere	4	BL	300	376	574
Dome-cylinder	2	B	449	1259	630
Dome-cylinder	2	BL	416	795	649
Dome-cylinder	3	B	394	664	627
Dome-cylinder	3	BL	373	487	658
Dome-cylinder	4	B	347	472	610
Dome-cylinder	4	BL	353	452	630
Cylinder	2	B	452	768	571
Cylinder	2	BL	353	802	588
Cylinder	3	B	413	1025	687
Cylinder	3	BL	327	596	700
Cylinder	4	B	439	949	695
Cylinder	4	BL	289	492	578

A single common mask (the union of all seven candidate marker sites, fixed 4.0 mm radius) was applied to every design, every marker size and both modalities. The initial pose was taken from the principal axes of the masked point clouds, so no marker coordinate and no marker-bearing coordinate frame entered the registration; the markers served only as independent verification targets. Group medians: 385, 382 and 350 μm at 2, 3 and 4 mm ($p = 0.653$); B 394, BL 327 μm ($p = 0.013$). The method × size interaction against the marker-based residual of Table S3 was tested on the paired design-level values (mixed-effects model with

design as a random intercept, $\chi^2 = 10.78$, df = 2, p = 0.005; Kruskal–Wallis on the within-design paired difference, p = 0.055; median paired ratio 1.34, 1.31 and 0.93 at 2, 3 and 4 mm).

Table S5. Markerless residuals at marker positions exceeding local-error thresholds (270 markers).

Threshold (μm)	Markers exceeding	Total	Percent
500	40	270	14.8
750	13	270	4.8
1000	6	270	2.2
1500	4	270	1.5
2000	4	270	1.5

Reported CBCT–intraoral-scan registration errors and the deviations quoted for guided implant surgery are generally of sub-millimeter magnitude, so a local error beyond 1000 μm was taken as the reference level of clinical concern; counts at neighbouring thresholds are given so that the summary does not rest on a single cut-off or on the single largest case (maximum 2847 μm).
